# Development and quantitative assessment of deep learning-based image enhancement for optical coherence tomography

**DOI:** 10.1186/s12886-022-02299-w

**Published:** 2022-03-26

**Authors:** Xinyu Zhao, Bin Lv, Lihui Meng, Xia Zhou, Dongyue Wang, Wenfei Zhang, Erqian Wang, Chuanfeng Lv, Guotong Xie, Youxin Chen

**Affiliations:** 1 Department of Ophthalmology, Peking Union Medical College Hospital, Chinese Academy of Medical Sciences, Beijing, 100730 China; 2 Key Lab of Ocular Fundus Diseases, Chinese Academy of Medical Sciences, Beijing, 100730 China; 3 Ping An Healthcare Technology, 9F Building B, PingAn IFC, No.1-3 Xinyuan South Road, Beijing, 100027 China; 4 Ping An Health Cloud Company Limited, Shenzhen, 518000 China; 5 Ping An International Smart City Technology Company Limited, Shenzhen, 518000 China

**Keywords:** Optical coherence tomography, Deep learning, Image enhancement, Quantitative assessment

## Abstract

**Purpose:**

To develop a deep learning-based framework to improve the image quality of optical coherence tomography (OCT) and evaluate its image enhancement effect with the traditional image averaging method from a clinical perspective.

**Methods:**

359 normal eyes and 456 eyes with various retinal conditions were included. A deep learning framework with high-resolution representation was developed to achieve image quality enhancement for OCT images. The quantitative comparisons, including expert subjective scores from ophthalmologists and three objective metrics of image quality (structural similarity index measure (SSIM), peak signal-to-noise ratio (PSNR) and contrast-to-noise ratio (CNR)), were performed between deep learning method and traditional image averaging.

**Results:**

With the increase of frame count from 1 to 20, our deep learning method always obtained higher SSIM and PSNR values than the image averaging method while importing the same number of frames. When we selected 5 frames as inputs, the local objective assessment with CNR illustrated that the deep learning method had more obvious tissue contrast enhancement than averaging method. The subjective scores of image quality were all highest in our deep learning method, both for normal retinal structure and various retinal lesions. All the objective and subjective indicators had significant statistical differences (*P* < 0.05).

**Conclusion:**

Compared to traditional image averaging methods, our proposed deep learning enhancement framework can achieve a reasonable trade-off between image quality and scanning times, reducing the number of repeated scans.

## Introduction

Optical coherence tomography (OCT) is a noninvasive imaging technique which has been widely used to obtain a cross-sectional retinal structure in ophthalmology [1]. It brings a new revolution for the diagnosis of ophthalmic diseases [2]. However, image quality can affect the clinical interpretation and many efforts have been made to enhance OCT image quality [3]. Although spectral-domain OCT (SD-OCT) has significant improvement in scanning speed, axial resolution and signal-to-noise ratio [1, 3], the image quality still inevitably suffers from speckle noise brought by OCT intrinsic imaging principle [3]. For the evaluation of retinal disease, the quality of OCT images is of great importance for accurate retinal disease detection. Taking paracentral acute middle maculopathy (PAMM) and retinal angiomatous proliferation (RAP) for examples, only OCT images with high quality and proper enhancement could facilitate an accurate diagnosis. Currently, the common enhancement method is image averaging, which is performed by obtaining multiple B-scan frames at the same location and then averaging them [4–6]. It is firstly used on time-domain OCT to achieve better visualization of retinal layers [4]. Then Sakamoto et al. applies image averaging on SD-OCT images and achieved significant improvement in both objective metrics and the ophthalmologists’ ability of distinguishing retinal lesions [5]. With subjective scoring, image averaging has been proved to be a statistically significant benefit for assessing the external limiting membrane (ELM) and outer nuclear layer (ONL) [7]. Then a detailed comparative experiment indicates that OCT image quality improves with an increase in the number of frames averaged, and the optimal number is 20 while minimizing the examination time and maximizing the image quality [8]. However, image averaging requires multiple repeated scans at the same location while the subject is instructed to maintain steady fixation. Besides the increase of examination time, its performance is also affected by imperfect registration among repeated scans [9].

Recently, deep learning has achieved significant progress in medical images [10]. In the ophthalmic images, in addition to identifying disease features [11], there are some applications for OCT image enhancement [12–15]. Conventional neural networks [13–15] and generative adversarial networks [12] are selected to achieve OCT image enhancement as the image-to-image translation from low quality to high quality. Enhancement performance is then evaluated by objective quantitative metrics such as peak signal-to-noise ratio (PSNR) and structural similarity index measure (SSIM) [13, 16]. Although these studies have shown deep learning can enhance image quality while reducing scanning times, there are some unsolved problems involving enhancement patterns and performance evaluation. Firstly, all existing studies perform image enhancement based on a single B-scan image and do not investigate whether image quality can improve continually while network inputs become multiple frames [12–15]. Secondly, performance evaluation is only performed with objective metrics rather than the subjective assessment from a clinical perspective, which has been conducted for image averaging [5, 8].

In this study, we propose a deep learning framework to enhance OCT images with multiple frame inputs, and compare its performance with traditional image averaging using objective metrics and subjective expert assessment. We perform our experiments on OCT images acquired from healthy individuals and patients with retinal diseases.

## Materials and methods

This observational study used OCT images from Peking Union Medical College Hospital, which were collected from 447 participants between August 2019 to March 2020. There were 196 healthy individuals and 251 patients with retinal diseases. Prior to the OCT imaging, all patients underwent eye examination on a slit lamp along with an examination of the posterior segments using an ophthalmoscope. The exclusion criteria included patients with non-retinal conditions (i.e. glaucoma), media opacity which unable OCT imaging to be performed, extreme ametropia and abnormal anterior segment (i.e. corneal haze, significant cataract). The study was approved by the Ethics Committee of Peking Union Medical College Hospital, Chinese Academy of Medical Sciences (No. HS-2174). The whole process adhered to the tenets of the Declaration of Helsinki, and written informed consent was obtained from each participant.

### OCT scanning and image averaging

**Fig. 1:**
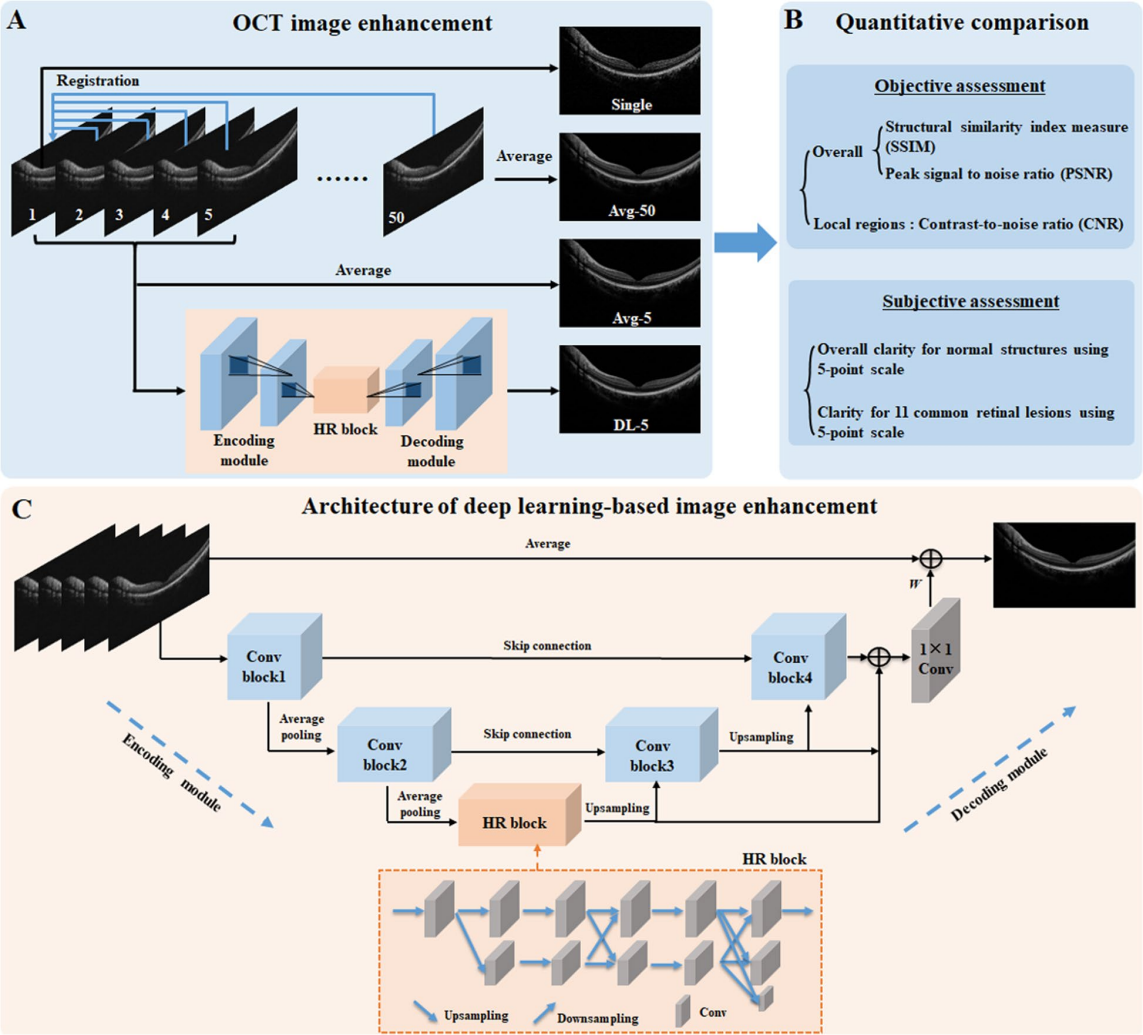
The schematic diagram of traditional image averaging and the proposed deep learning-based OCT image enhancement method (**A**), and the quantitative comparisons for their generated images (**B**)

All images were scanned by a commercial SD-OCT system (Mocean 3000 plus, Shenzhen Moptim Imaging Technique Co. Ltd., China, http://www.moptim.com/). 50 OCT frames were horizontal B-scans collected at the same position across the center of the macular region. The scanning range was 12 × 12 mm and the image resolution was 4000 × 860. Before image averaging, we registered the original images by optimizing the mutual information between pixels in each single B-scan image. The scanning frequency of our OCT camera is 80,000 Hz and 20 OCT images per second can be obtained from the same position. The scanning speed is relatively fast and the eye movement can be considered as uniform movement within 1/20 s, we therefore only performed rigid registration among them for most normal situations. The one with the strongest signal-to-noise ratio in scanned images was selected as the reference image. The rest images were rotated and rigidly changed to align with the baseline image. Then the enhanced image was obtained by averaging all the aligned images (Fig. 1). We recorded the time of image scanning and alignment based on system time.

We excluded the OCT images with poor image quality caused by poor fixation. The definition of poor fixation was that the correlation coefficient between the registered image and the reference image is lower than 70% of the autocorrelation coefficient of the reference image. All the registration steps were implemented using the software provided by the OCT manufacturer of Moptim. Finally, 815 sets of OCT images were collected, and each set corresponded to the collected images from each eye, consisting of 50 aligned OCT frames and 1 enhanced OCT image (called Avg-50) averaged from 50 aligned frames. We considered Avg-50 image as gold standard for the best quality. There were 359 image sets from normal eyes and 456 image sets from abnormal eyes. Among them, 610 sets (264 normal eyes and 346 abnormal eyes) were used for training deep learning enhancement model and 205 sets (95 normal eyes and 110 abnormal eyes) were used for performance evaluation.

### Deep learning image enhancement

We developed a deep learning architecture for OCT image enhancement as shown in Fig. 1. It was constructed based on the idea of U-net [17] which contained an encoding module and a decoding module. The encoding module was implemented by two contracted convolution blocks, while the decoding module included two extended convolution blocks. To enhance the ability to express image details, we added the high-resolution representation block [18] after the encoding module, and a side path was integrated to contact the features of decoding modules with different resolutions. Feature maps were extracted from the input OCT images through the encoding convolution blocks, and then were gradually restored to a high-definition image through the decoding convolution blocks. In particular, our network inputs were designed as multiple OCT frames in order to acquire more original scanned information during image enhancement. The number of network input ranged from 1 to 20. One reason of choosing 1 to 20 is the consideration of computation power, another reason is that a previous detailed comparative study has indicated that the optimal number was 20 while minimizing the examination time and maximizing the image quality [8]. Finally, the network output, namely the enhanced OCT image, was obtained by performing a weighted sum of the averaged multiple inputs and the restored feature maps after the decoding module.

We used 460 sets (200 normal eyes and 260 abnormal eyes) from 610 sets as the training dataset, and the rest 150 sets (64 normal eyes and 86 abnormal eyes) were used as the validation dataset. Each set composed 50 aligned frames and an averaged image called Avg-50. The subsets of frames were selected as network inputs, while their corresponding Avg-50 was considered as network output. Such subsets were selected randomly from the whole 50 frames with many times to achieve offline data augmentation. During training, the cross-entropy and SSIM [16] were referred to the optimization loss. Adam optimizer was used to train the established network, and Xavier algorithm was used to initialize model weights. The learning rate was initialized to 0.001. If the loss did not decrease within 10 epochs, we applied a strategy that automatically decayed the learning rate by factor of 0.9. All inputs and outputs were normalized to a range between 0 and 1. The deep learning network was implemented in Python (https://www.python.org/) with Keras (https://keras.io/). And all the training process was performed on an NVIDIA Tesla P100 GPU.

### Quantitative assessment

We performed quantitative assessments from objective and subjective perspectives. Three objective metrics were applied: SSIM [16] and PSNR [12] for the entire image and contrast-to-noise ratio (CNR) for selected regions of interest (ROIs) (Fig. 1B). And subjective assessment was performed by four ophthalmologists on 205 image sets.

**Fig. 2:**
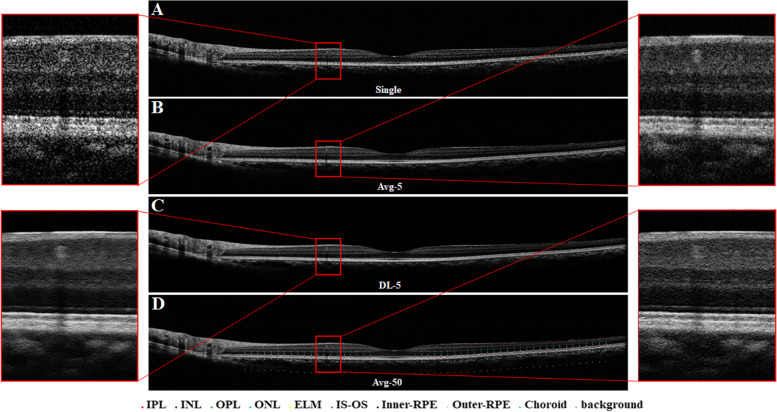
A representative OCT image scanned from a healthy eye. **A** is original single-frame; **B** is the enhanced OCT image (Avg-5) by averaging 5 frames; **C** is the enhanced OCT image (DL-5) generated by deep learning method with 5 frames; **D** is the enhanced OCT image (Avg-50) by averaging 50 frames. Regions with red rectangle are zoomed for visual examination. As shown in (**D**), in order to measure the CNR, we marked manually 50 pairs of regions of interest with size 4 × 4 pixels in different retinal tissues

We calculated SSIM and PSNR between a gold standard and each enhanced image by different methods. Besides, we paid special attention to the enhancement performance in some important retinal structures. Previous studies have applied CNR to evaluate the contrast between different tissue layers [5, 8, 13]. It was defined as $\mathrm{CNR}=\frac{h-l}{\sqrt{\delta_h^2+{\delta}_l^2}}$, where *h* and *l* are the mean pixel intensity, and ${\delta}_h^2$ and ${\delta}_l^2$ are the variance of the ROIs from the high-reflection layers and the low-reflection layers, respectively. The ROIs were selected from the following retinal tissues: (1) inner plexiform layer (IPL); (2) inner nuclear layer (INL); (3) outer plexiform layer (OPL); (4) ONL; (5) ELM; (6) inner–outer segments junction (IS-OS); (7) inner retinal pigment epithelium (Inner-RPE); (8) outer retinal pigment epithelium (Outer-RPE); (9) choroid; and (10) background. 50 pairs of ROIs with 4 × 4 pixels were manually marked by an ophthalmologist from the corresponding tissues at equal intervals (Fig. 2D). The high-reflection layers were IPL, OPL, ELM, IS-OS, Outer-RPE and choroid, and the low-reflection layers were INL, OTL, Inner-RPE, and background. Therefore, we calculated the CNR values in pairs of IPL/INL, OPL/ONL, ELM/ONL, IS-OS/Inner-RPE, Outer-RPE/Inner-RPE, and choroid/background.

For subjective assessment, each set included an original single frame, enhanced image with the traditional averaging, enhanced image with deep learning and Avg-50 image. They were displayed for assessment simultaneously. The top one and the bottom one were the original single frame and Avg-50 image, respectively. To avoid possible misleading, the enhanced images with traditional averaging and deep learning were displayed in the middle random order.

Before the assessment, all the reviewers completed a training course to learn the scoring criteria with 10 images. They were instructed to assign scores for comparative image quality using the 5-point scale. 1 point was assigned to a single frame as a lower limit, while 5 points were assigned to Avg-50 image as an upper limit. And 2, 3, 4 points were scored to different enhanced images according to their visual observation compared to upper and lower limits. For normal OCT images, ophthalmologists evaluated the overall clarity of anatomical structures. For abnormal images, the reviewers also rated different scores for different lesions, respectively. There were 11 common retinal lesions shown in Table 1. When multiple lesions with the same type were present in one image, we would make a comprehensive assessment for each type. We applied the weighted Cohen’s kappa statistics to assess the degree of agreement within individual graders. The average scores of four reviewers were used as final subjective scores. Then paired sample t-tests were applied to compare the scores between each pair of enhanced images for retinal anatomical structures and different retinal lesions, respectively. *P* < 0.05 was considered as the statistical significance.

**Table 1:** The statistics of retinal lesions included in 110 abnormal eyes for quantitative comparison of image enhancement

Lesion	No. of Images
IRF	21
ARPE	20
Choroid change	20
SRF	17
ERM	16
CNV	13
SHRM	12
Hyper-reflective Foci	11
Macular Hole	11
PED	10
ME	10

ARPE Atrophy of retinal pigment epithelium, *CNV* Choroidal neovascularization, *ERM* Epiretinal membrane, *IRF* Internal retinal fluid, *ME* Macular edema, *PED* Pigment epithelium detachment, *SHRM* Subretinal hyperreflective material, *SRF* Sub-retinal fluid

## Results

We investigated the influence of a different number of inputs on image enhancement results with traditional averaging and our proposed method. Figure 4A, B showed the relationships between quantitative metrics (SSIM/PSNR) and the number of frames used in two methods. With the increase of frame count from 1 to 20, SSIM values were increased from 0.83 to 0.97 for deep learning method, and from 0.47 to 0.95 for traditional averaging. Meanwhile, PSNR values were increased from 30.23 to 40.1 for deep learning method, and from 23.93 to 39.4 for averaging method. We could observe that deep learning method obtained higher SSIM and PSNR values than averaging method while importing the same number of frames. Based on these objective metrics, we could infer that deep learning method had a better ability to improve OCT image quality than traditional averaging. Of note, the rapid growth of SSIM occurred while the number of frames was less than 5 for both methods. Deep learning method with 5 frames could achieve the comparable SSIM with the averaging method with 16 frames. In order to investigate the performance of image quality with few scanned images as possible, we therefore selected 5 frames as inputs of different methods for following quantitative comparisons from both subjective and objective perspectives.

**Fig. 3:**
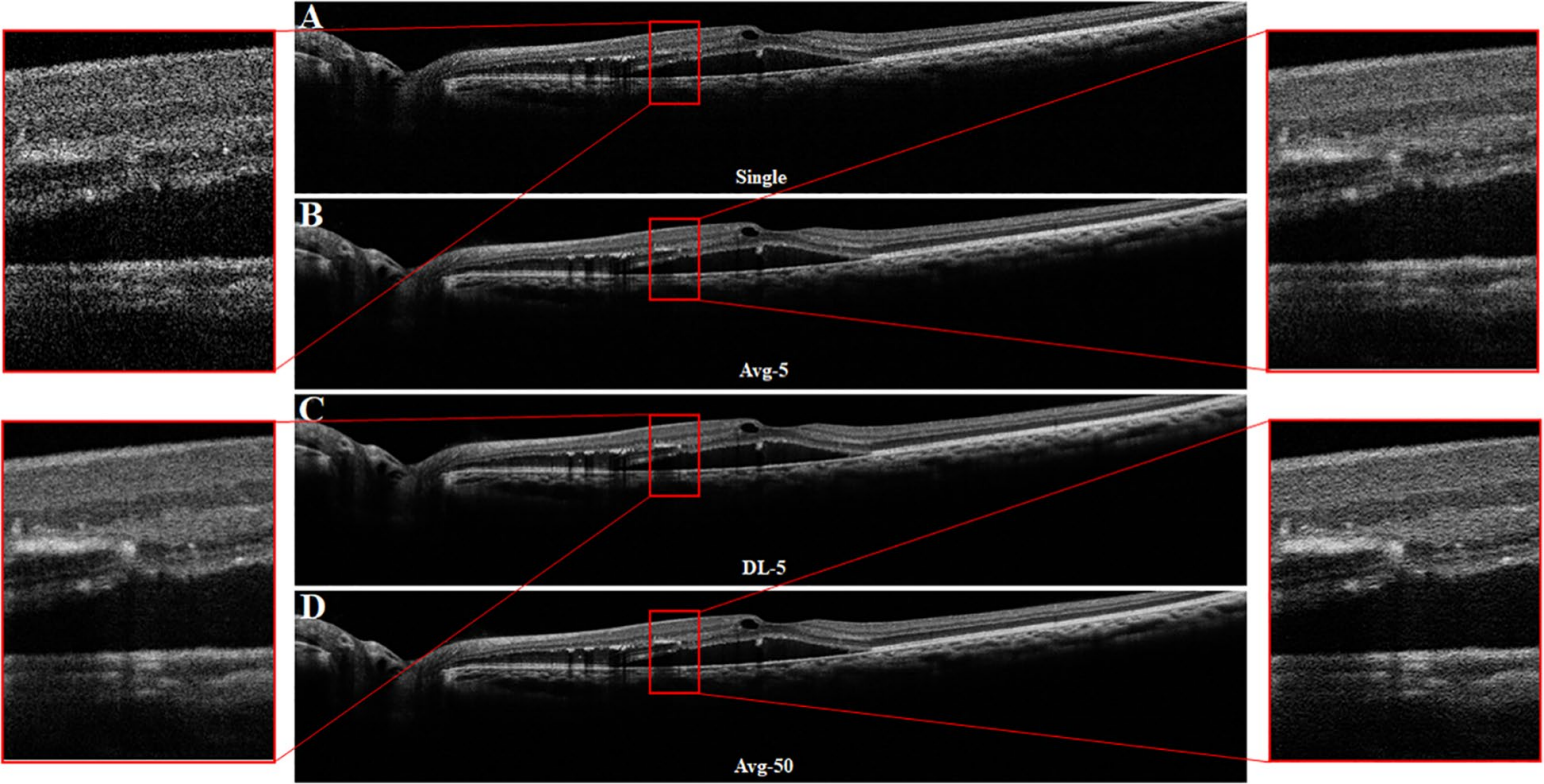
A representative OCT image scanned from an abnormal eye with hyper-reflective foci, internal retinal fluid and sub-retinal fluid. **A** is original single-frame; **B** is the enhanced OCT image (Avg-5) by averaging 5 frames; **C** is the enhanced OCT image (DL-5) generated by deep learning method with 5 frames; **D** is the enhanced OCT image (Avg-50) by averaging 50 frames. Regions with red rectangle are zoomed for visual examination

**Fig. 4:**
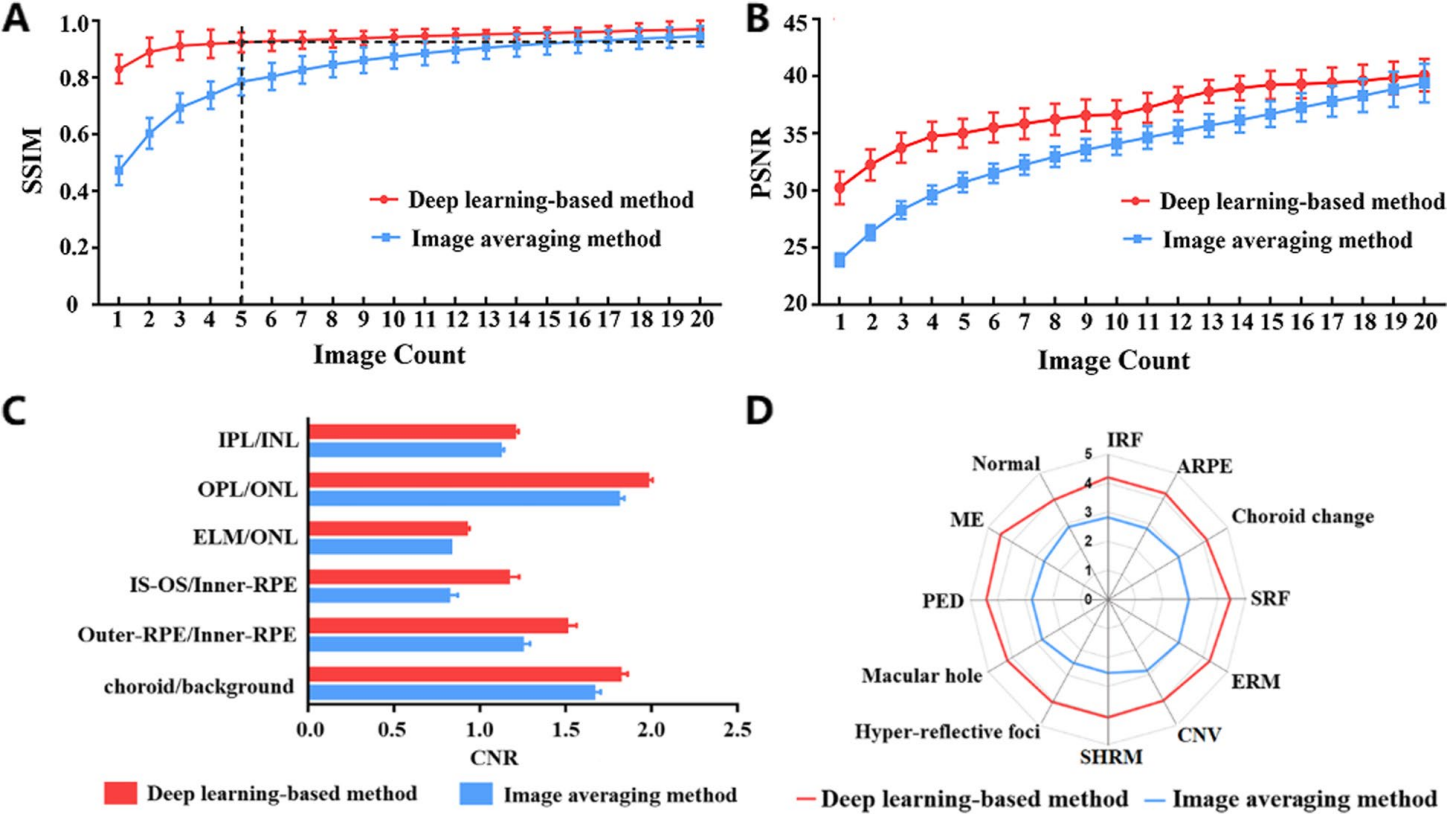
The results of quantitative assessment for image enhancement. **A** and **B** are the relationships (means and covariances in 205 images) between quantitative metrics and number of frames used in traditional averaging (blue lines) and deep learning method (red lines). **C** is CNR comparisons (means and covariances in 205 images) between image averaging (blue bar, Avg-5) and deep learning method (red bar, DL-5). **D** is subjective scoring (means values in 205 images) for image quality enhanced by traditional averaging (blue line, Avg-5) and deep learning method (red line, DL-5) for normal retinal structure and retinal lesions

Figures 2 and Fig. 3 showed the representative results scanned from a normal eye and an abnormal eye, respectively. The compared images included the original single frame, the enhanced images by averaging 5 frames (Avg-5) and 50 frames (Avg-50), and the enhanced image by deep learning method with 5 frames (DL-5). Compared to a single frame, all enhanced images (Avg-5, DL-5 and Avg-50) had the clearer retinal layer structures and anatomical abnormalities such as hyper-reflective foci, internal retinal fluid and sub-retinal fluid. DL-5 had a more similar visualization with Avg-50 relative to Avg-5. These visual findings were confirmed by objective metrics. In the evaluation dataset of 205 images, the averaged SSIM and PSNR values were both higher in our method than those in traditional averaging (0.92 ± 0.03 and 0.78 ± 0.04 for SSIM of DL-5 and Avg-5, respectively whilst the PSNR for DL-5 and Avg-5 were 35.01 ± 1.25 and 30.70 ± 0.85, respectively). Figure 4C showed the average CNR performance for paired ROIs. For all ROIs, there were significant statistically differences between our proposed method and the traditional method (DL-5 vs Avg-5: 1.23 ± 0.04 vs 1.11 ± 0.06 for IPL/INL; 1.97 ± 0.06 vs 1.80 ± 0.07 for OPL/ONL; 0.92 ± 0.02 vs 0.83 ± 0.04 for ELM/ONL; 1.17 ± 0.12 vs 0.82 ± 0.10 for IS-OS/Inner-RPE; 1.51 ± 0.11 vs 1.25 ± 0.08 for Outer-RPE/Inner-RPE; and 1.81 ± 0.08 vs 1.66 ± 0.07 for choroid/background).

The Cohen’s Kappa value was 0.71 for the subjective scoring, which meant that four reviewers reached the substantial agreement according to Landis and Koch’s scale (Landis et al. 1977). Figure 4D showed the averaged values of subjective scoring for DL-5 and Avg-5, respectively. The results indicated that subjective scores were all higher in deep learning method than in traditional averaging for normal structure and lesions. There were all significant statistical differences (*P* < 0.05). For normal retinal structure, the subjective scores were 4.50 ± 0.27 for our method and 2.65 ± 0.59 for traditional averaging. For retinal lesions, the average scores of deep learning method and averaging method were 4.03 ± 0.32 vs 2.84 ± 0.41 for choroidal neovascularization (CNV), 4.14 ± 0.53 vs 2.97 ± 0.42 for choroid change, 4.27 ± 0.41 vs 2.98 ± 0.60 for epiretinal membrane (ERM), 4.07 ± 0.61 vs 2.53 ± 0.89 for hyper-reflective foci, 4.20 ± 0.48 vs 2.82 ± 0.78 for internal retinal fluid (IRF), 4.50 ± 0.27 vs 2.65 ± 0.59 for macular edema (ME), 4.21 ± 0.51 vs 2.76 ± 0.57 for macular hole, 4.41 ± 0.46 vs 2.76 ± 0.68 for pigment epithelium detachment (PED), 4.20 ± 0.45 vs 2.82 ± 0.53 for atrophy of retinal pigment epithelium (ARPE), 4.07 ± 0.31 vs 2.54 ± 0.58 for subretinal hyperreflective material (SHRM) and 4.45 ± 0.57 vs 2.95 ± 0.65 for sub-retinal fluid (SRF), respectively.

In our experimental OCT device, it took an average of 1.7 min to scan and generate the enhanced image (Avg-50) with the best image quality by averaging 50 frames. Meanwhile, it took an average of 30 s to scan and align 5 frames, and an additional time of 50 milliseconds to generate the enhanced image (DL-5) through a deep learning network. Therefore, our deep learning method only required about 30.5 s to generate an enhanced image with acceptable image quality, which reduced the scanning time drastically by more than 3 folds.

## Discussion

We presented a new deep learning algorithm with high-resolution representation to achieve OCT image quality enhancement, and performed the quantitative comparison with image averaging. Dataset for comparison included normal images and abnormal OCT images with retinal lesions. The comparison results were reported on two levels: objective metrics of image quality and subjective scores from ophthalmologists. We revealed that image quality would be better with the increase of the number of input frames in both image enhancement methods. Our proposed deep learning method could improve OCT image quality more effectively than traditional averaging while importing the same number of frames, which was assessed by both objective and subjective indicators. Our deep learning model of 5 frames achieved a comparable image enhancement with the averaging method of 10 frames and even more. Therefore, our method could enhance the OCT image while reducing the scanning time, which had an obvious advantage over traditional averaging methods. The Kappa value 0.71 was of substantial agreement in our study, while for ERM and normal cases, the graders achieved the lowest agreement. This was reasonable as the features of ERM and normal retina could all be clearly displayed on OCT images, thus the evaluation between the graders might not be unified.

Compared to previous deep learning-based methods used in OCT image enhancement [12–15], our method has two innovations, one is network input, another is network architecture. Firstly, most previous methods only selected a single B-scan frame as network input, while our method attempted multiple B-scan frames. In theory, multiple frames provided more information than a single frame that could be a benefit for image enhancement [5]. Our experimental results demonstrated that better image quality was obtained with the increase of frame number. Secondly, our network architecture included a high-resolution representation block [18]. Using this network architecture, we can utilize more image detailed information effectively to enhance OCT image quality while suppressing image noise.

Most previous deep learning studies only performed the objective assessment for image quality enhancement [12–15], and did not conduct the subjective expert assessment from a clinical perspective. Here, our quantitative comparisons included not only objective assessment and but also subjective assessment for both normal eyes and abnormal eyes. Compared to traditional averaging, we could find that our deep learning achieved higher similarity to the gold standard with fewer frames (Fig. 4A). Similar assessment results for the whole image could be found by PSNR (Fig. 4B). Furthermore, the local assessment with CNR illustrated that deep learning method had more obvious tissue contrast enhancement than averaging method (Fig. 4C). The most enhanced CNR occurred in the pairs of IS-OS/Inner-RPE and Outer-RPE/Inner-RPE. Previous studies demonstrated that the multiple frame averaging method and single frame based deep learning denoising method could increase CNR values in various retinal tissues [5, 13], which were consistent with our experimental results. Higher CNR represents more distinguishable and recognizable among different retinal cell layers. In addition to helping clinical reading, such image quality enhancement could improve the accuracy of segmentation for retinal layer boundaries [19]. For subjective assessment, the average scores assigned by four experienced ophthalmologists illustrated our method achieved better image quality than traditional averaging (Fig. 4D). When 1 point was defined to single frame and 5 points were defined to Avg-50, the average scores were between 4 points and 5 points for deep learning enhanced images (DL-5) and were between 2 points and 3 points for enhanced images by averaging method (Avg-5). Particularly, we found that the scores of some low-reflection retinal lesions (e.g. ME, PED, and SRF) were higher than that of other lesions by deep learning method. These subjective assessments demonstrated that our method provided better visualization results than averaging method under various retinal conditions.

Our method significantly reduced the number of scanning frames at the same location while enhancing OCT image quality. For patients who have difficulty in maintaining pupil dilation for a long time, our method can reduce the patient’s cooperation requirements, thereby avoiding additional clinical auxiliary support. Another advantage of fewer repeat scans at the same location is that they reduce the effect of eye movement on image quality. Although an eye tracking device has been used, the effects of eye movement cannot be completely eliminated and registration errors will occur [9]. A direct and effective way is to reduce the number of repeated scans, because patients can better control eye movements in a shorter time. With our method, we can reduce the number of scans at the same location and provide more capabilities to achieve a wider range of 3D volumetric scan.

Several limitations need to be strengthened in the future. Firstly, our method was designed to improve the image quality while reducing the number of repeated scans, and its performance was compared with traditional image averaging methods. It cannot be used to deal with the problems of image quality evaluation and quality control (e.g. signal loss, mirror artifact and motion artifact). Our previous study [20] and other researchers [21] have applied deep learning methods to achieve OCT image quality evaluation and automatic quality control. Further work includes how to solve image enhancement and quality control at the same time. Secondly, we evaluated the image quality based on subjective and objective indicators directly. Poor image quality could lead to incorrect tissue measurement [22] and then incorrect clinical decisions [23]. Assuming the improved visualization of retinal layers and anatomical abnormalities after image enhancement, it might bring some benefits for automatic analysis, including more accurate segmentation and diagnosis for retinal diseases [24]. Therefore, we can perform such indirect comparative experiments of different image enhancement methods to evaluate their effectiveness. Thirdly, we validated the effectiveness of our deep learning-based image quality enhancement method on Mocean 3000 plus, since we could only obtain the original scanned frames from this device with the technical help of the manufacturer. Although the trained deep learning model cannot be used to other OCT devices, we think our proposed image enhancement algorithm can be generalized when we collect the dataset and retrain the model from other OCT devices with the same imaging principle of SD-OCT. At last, OCT angiography (OCTA) can provide a highly detailed view of the retinal microvascular morphologic features noninvasively [25]. Recent studies have demonstrated that image averaging is also a powerful tool for enhancing OCTA image quality [6, 26–28]. A further step of our research could be an application of the similar deep learning algorithm to verify the potential improvement of the quality of OCTA images.

In summary, our deep learning algorithm achieved better image enhancement for retinal tissues and their abnormal changes than the traditional image averaging method while importing the same number of scanned OCT frames. Our quantitative assessment suggests that a deep learning enhancement framework can improve OCT image quality for clinical diagnosis with less scanning times.

## Data Availability

The datasets used and/or analyzed during the current study are available from the corresponding author on reasonable request.

## Abbreviations

ARPE: Atrophy of Retinal Pigment Epithelium
CNV: Choroidal Neovascularization
CNR: Contrast-to-Noise Ratio
ELM: External Limiting Membrane
ERM: Epiretinal Membrane
IPL: Inner Plexiform Layer
INL: Inner Nuclear Layer
IS-OS: Inner–Outer Segments Junction
Inner-RPE: Inner Retinal Pigment Epithelium
IRF: Internal Retinal Fluid
ME: Macular Edema
OCT: Optical coherence tomography
OPL: Outer Plexiform Layer
ONL: Outer Nuclear Layer
Outer-RPE: Outer Retinal Pigment Epithelium
PSNR: Peak Signal-to-Noise Ratio
PED: Pigment Epithelium Detachment
ROIs: Regions of Interest
SD-OCT: Spectral-Domain Optical Coherence Tomography
SNR: Signal-to-Noise Ratio
SSIM: Structural Similarity Index Measure
SHRM: Subretinal Hyperreflective Material
SRF: Sub-Retinal Fluid
